# Modelling of pH-dependence to develop a strategy for stabilising mAbs at acidic steps in production

**DOI:** 10.1016/j.csbj.2020.03.002

**Published:** 2020-03-10

**Authors:** Max Hebditch, Ryan Kean, Jim Warwicker

**Affiliations:** a School of Chemistry, Faculty of Science and Engineering, University of Manchester, Manchester Institute of Biotechnology, 131 Princess Street, Manchester M1 7DN, UK; b School of Biological Sciences, Faculty of Biology, Medicine and Health, University of Manchester, Manchester Institute of Biotechnology, 131 Princess Street, Manchester M1 7DN, UK

**Keywords:** Biophysics, Biopharmaceuticals, Antibodies, Purification, Acid-stability, Electrostatics

## Abstract

Engineered proteins are increasingly being required to function or pass through environmental stresses for which the underlying protein has not evolved. A major example in health are antibody therapeutics, where a low pH step is used for purification and viral inactivation. In order to develop a computational model for analysis of pH-stability, predictions are compared with experimental data for the relative pH-sensitivities of antibody domains. The model is then applied to proteases that have evolved to be functional in an acid environment, showing a clear signature for low pH-dependence of stability in the neutral to acidic pH region, largely through reduction of salt-bridges. Interestingly, an extensively acidic protein surface can maintain contribution to structural stabilisation at acidic pH through replacement of basic sidechains with polar, hydrogen-bonding groups. These observations form a design principle for engineering acid-stable proteins.

## Graphical abstract

**Figure:**
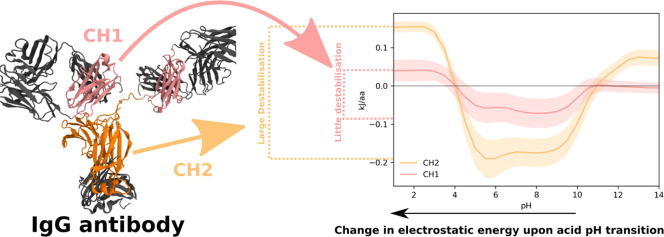

## 1 Introduction

The promise of therapeutic antibodies relies on the ability of the pharmaceutical industry to develop large scale manufacturing processes that can produce safe, economic and reproducible formulations. However, these biopharmaceutical production processes are limited by non-specific interactions between the proteins, which can lead to reversible or irreversible association. At best, these association potentials can lead to batch to batch inconsistencies, but more worryingly they can lead to the abandonment of promising therapeutic leads due to an inability to produce a stable formulation, the loss of target specificity or the development of immunogenicity amongst other issues. One of the major driving forces for reversible association is the solution conditions used to purify monoclonal antibodies, in particular the requirement for low pH solution steps. Currently, most therapeutic mAbs are produced using a similar pipeline. Antibodies are normally expressed using mammalian expression systems, usually CHO cells [1], once secreted the antibodies are then purified using chromatographic and membrane filtration steps to remove impurities. Protein A chromatography was one of the first protein purification processes developed [2], and is still the state of the art for the manufacture of biotherapeutic antibodies [3], [4] as it is highly effective, resulting in >98% purity in a single step [5]. It is also highly stable, retaining specificity over multiple uses [6]. However, the use of protein A necessitates the use of a low pH buffer to elute the protein from the column, and this acid titration can induce antibody aggregation leading to protein formulation issues [7], [8], [5]. It has also been suggested that the column itself may contribute to aggregation [9]. Following protein A purification, antibodies expressed from mammalian sources also require a low pH hold for viral inactivation which can lead to further aggregation [7]. As well as aggregation, it has been observed that exposure to acidic pH can cause polyreactivity, which may be part of the natural immune response, but is an issue for developing specifically targeted therapeutics [10], [11].

In light of this, attempts have been made to modify protein A to allow the use of less acidic buffers [12], [13], [14], to optimise the resin [15] and washes [16], or develop alternative chromatographic processes [17]. The development of alternative purification techniques such as controlled conductivity at low pH [18] and engineered proteins with a calcium dependent binding for IgG [19] has also been discussed. These techniques however do not remove the need for a low pH hold for viral inactivation, instead alternative techniques have been suggested [20], [21]. The use of excipients like arginine to help minimise damage from low pH treatments has also been reported [22].

**Fig. 1:**
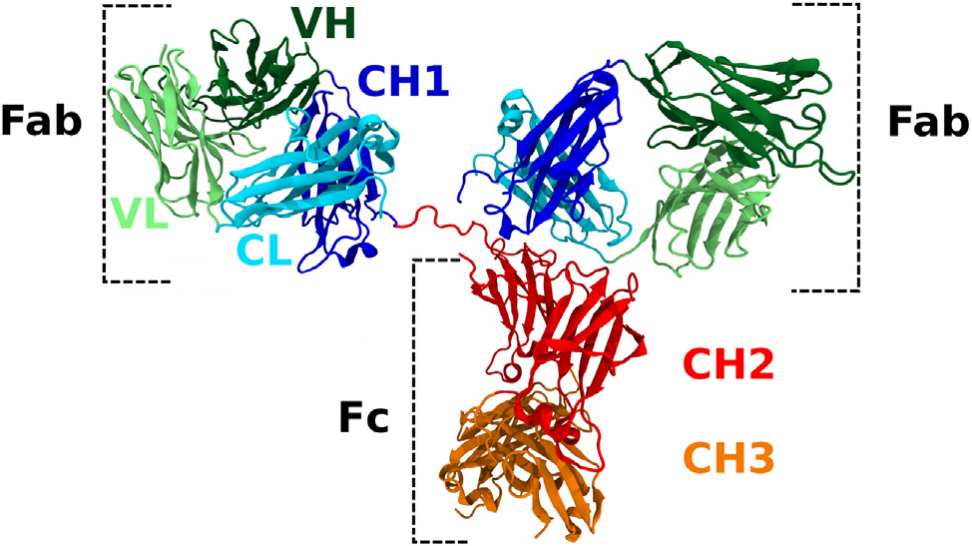
Example schematic of a IgG (pdb id1hzh, [33]). Each IgG is comprised of two heavy chains (each containing a VH, CH1, CH2 and CH3) and two light chains (each containing a VL and CL).

Due to the requirements of protein A chromatography and viral inactivation, many reports have investigated antibody pH sensitivity and aggregation pathways [23], [24], [25], [26], [27], [28], [29], [30]. To better understand the cause of acid induced protein aggregation, research has focussed on understanding if aggregation is driven by specific domains within the antibody. The IgG is routinely divided into its two functional substructures, the Fab which contains the antigen binding region, and the Fc plays an important role in immunological signalling and activation (Fig. 1). These substructures can be further subdivided into individual domains. The Fab contains the variable heavy (VH) and variable light (VL) domains, named for their role in containing the hypervariable complementarity determining regions which bind the antigen, and the constant heavy (CH1) and constant light (CL) named for their conserved sequence. The Fc contains two further conserved heavy chain domains: CH2 and CH3.

The importance of electrostatics in stabilising proteins has been well studied in the literature, however, the contribution of ionisable group interactions to the stability of the folded state will differ between the charge environment that proteins have evolved for, in the case of antibodies physiological pH, and the low pH required for protein A chromatography and viral inactivation. A better understanding of the different role electrostatics play in stabilising the antibody at low and neutral pH is therefore important in order to develop more stable antibody formulations. In this work, we use Debye-Hückel calculations [31] to study the contribution of the ionisable group interactions to the folded state stability for the IgG, the Fab, Fc and all of the constituent domains. By studying the predicted response of the individual domains to low pH exposure, we attempt to determine which domains may be most sensitive to acid titration and thus drive low pH aggregation.

Previous work from our group has studied the difference in sequence and structure of the four Fab domains [32]. We determined that the CH1 domain has an unusual sequence composition described as being intrinsically disordered like, appearing to have little charge-charge stabilisation, and may instead be stabilised by its interaction with the CL domain. In this work, we report that the CH1 domain appears to be the least destabilised by acid titration, potentially due to its IDP-like characteristics, but more importantly our calculations suggest that the CH2 domain is the most destabilised at low pH, due to a large loss of ionisable interactions which are stabilising at neutral pH, but destabilising at low pH. This observation may provide insight for developing IgG therapeutics which will be resistant to aggregation in the low pH environment required as part of the industrial production of therapeutic mAbs. Through comparison with proteins that have evolved for functioning at low pH, we make suggestions for engineering strategies that could aid IgG domain stability in acidic conditions.

## 2 Method

### 2.1 IgG domain dataset acquisition

Structures for the Fab and Fc datasets were obtained from the protein data bank (PDB) [34]. The Fab domains were processed as discussed in Hebditch 2017 [32], in brief, the PDB was queried for the term “Fab”, including structures from human and mouse, as well as kappa and lambda light chains and following a number of quality control steps, the resulting structures were analysed for conserved interdomain sequence motif between the variable and constant domains and then cleaved, resulting in 333 unique Fabs and therefore 333 of each of the four Fab domains: VH, VL, CH, CL. Combined these domains also yield 333 heavy and light Fab chains. The Fc domains were obtained in a similar manner. The PDB was queried for the term “FC IgG1” which, excluding full length IgGs, returned 45 X-ray structures. These 45 chains were then aligned using Clustal Omega [35] and conserved motifs at the N and C terminus were identified for CH2 and CH3. For CH2 domains, the N’ motif was ‘PSVF’ and the C’ motif was ‘SK[AT]K’. For CH3 domains, the N’ motif was ‘GQPRE’ and the C’ motif was ‘LSL’. Retaining only structures with unique sequences resulted in 12 Fc PDBs and thus 12 CH2 and CH3 domain structures.

### 2.2 Acid environment proteins acquisition

A dataset of peptidases active at acidic pH was obtained from the MEROPS database [36]. The MEROPS database divides peptidases into families of homologous proteins. MEROPS A1 is a family of 27 endopeptidases with an aspartic active site, and are generally evolved to be most active in acidic environments. Structures were obtained from the PDB [34] for an acid-stable aspartic acid protease (pepsin, 3utl [37]), an acid-stable xylanase (1bk1, [38]), and an acid protease that has not evolved to be acid-stable (the BACE2 beta secretase 3zkq, [39]).

### 2.3 Electrostatic calculations

To assess the stability of the various IgG domains and acid environment proteins, we used electrostatics software previously described [31], [40], [41] and also available for free as a web application [42]. This software uses a Debye-Hückel calculation to calculate the pKas and thus the pH-dependent contribution to folded state stability. Using a Monte Carlo sampling of protonation we compare the stability of the unfolded state, where it assumed there are no interactions between ionisable groups, and the folded state. The folded state energy is defined in terms of kJ per amino acid to ensure a normalisation allowing direct comparison of different sized proteins and protein fragments. We used a continuum medium with a uniform relative dielectric constant of 78.4 (representing water) and an ionic strength of 0.15 M.

## 3 Results and discussion

### 3.1 Calculated pH-dependence of stability correlates with experiment

**Fig. 2:**
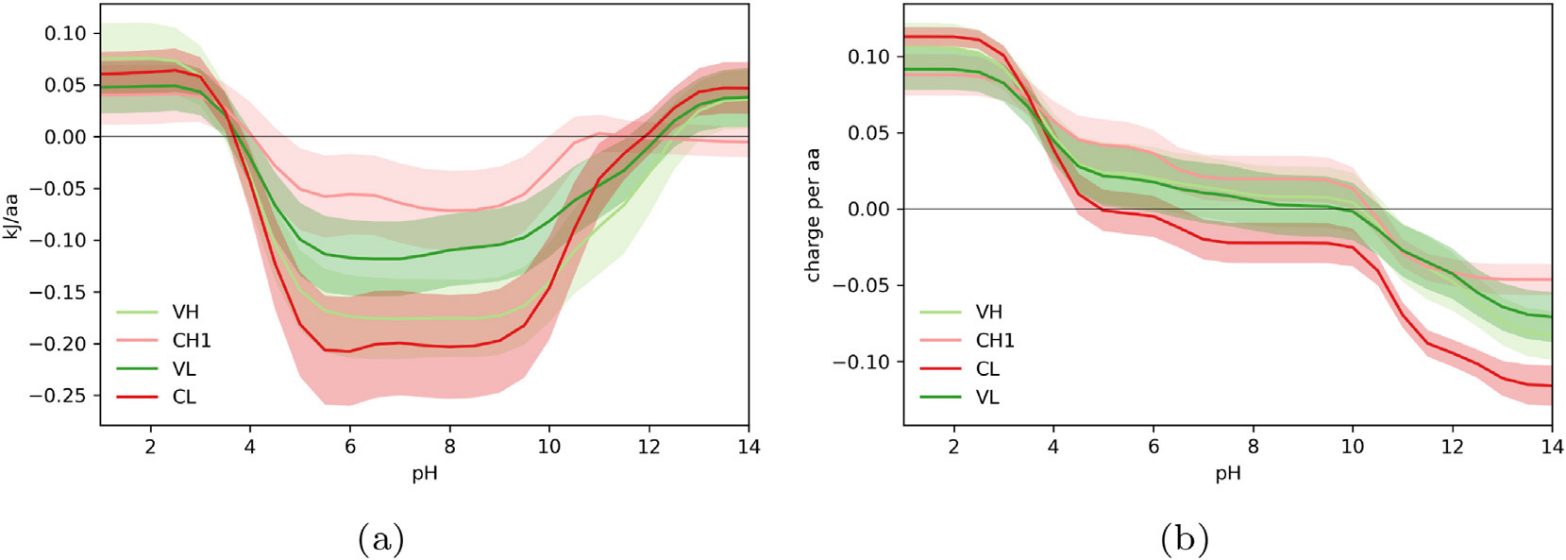
Calculated pH-dependence of folded state energy and charge for Fab domains. (a) The pH-dependent component of folded state energy is plotted against pH, showing average and 1 standard deviation spread for each domain type in the Fab dataset. (b) Predicted pH-titrated charge.

The Debye-Hückel approach calculates the electrostatic contribution to folded state stability from pH 1 to 14. Although a protein may not be stable at these extreme pH values, we use them (without accounting for any conformational change) to traverse the extent of ionisation states for the most important ionisable groups. For each pH value, a negative values indicate that the electrostatic contribution (from interactions between ionisable groups) will favour the formation of the folded state. Positive values suggest that the protein is not stably folded due to electrostatic forces alone, and will require an energetic input, or constraint, to maintain the folded state of the structure. We generally note a similar inverse bell distribution for most proteins, structure or domain interrogated, with each exhibiting the most electrostatic stability, evident from the most negative kJ/aa values, between pH 5 to 9, which is to be expected as IgGs have evolved to be stable at physiological pH (Fig. 2). At the more extreme pH values, less than pH 4 where the acidic residues are uncharged, and greater than pH 10 where the basic residues are uncharged, we observe positive kJ/aa values indicating that the proteins are less stable the further they are from physiological pH, where either the acidic or basic residues are uncharged and thus cannot contribute to the electrostatic stability of the folded state through ionic interactions.

The Fab substructure consists of four domains which appear to have different fold stability profiles. Of the four Fab domains, our calculations suggest that at physiological pH the CL domain has the greatest contribution from electrostatic forces to folded state stability and is therefore the most stable, followed by the VH and VL with CH1 exhibiting the lowest electrostatic stability (Fig. 2a). At low pH, the four domains exhibit similarly destabilising contributions from ionisation group interactions. As CH1 has the lowest loss of stability upon transition from neutral to acidic pH, we predict that the CH1 domain is the least destabilised by low pH conditions. CL in comparison loses a large degree of stabilising electrostatic force and is therefore more sensitive to changes in pH.

In terms of charge at neutral pH, the CL domain is calculated to have a negative charge, with the other three domains predicted to be positively charged. Of note, the VH and VL domains appear to exhibit very similar charge, but the CH1 and CL appear to have a difference in charge of around 0.04*e* per aa at physiological pH and changes in the charge for CH1 and CL appear to be closely related (Fig. 2b).

The difference in ionisation energy and charge suggests that within the Fab, the CH1 domain appears to exhibit surprising stability when transitioning from neutral to low pH, and there also appears to be a potentially compensatory relationship with the CL domain. In previous work from our group [32], we noted that the CH1 domain of the Fab sampled an amino acid composition subset and as a result was enriched for certain amino acids (P, S, T, V) and depleted for others (D, E, I, F, Q, R, Y). We described the CH1 domain as being IDP-like in the absence of the CL domain and this is supported by experimental work in the literature that notes that the CH1 domain is not stable in isolation [43], [44], but exhibited a very stable interaction with the CL domain. Similarly, γB-crystallins are highly soluble proteins with two domains that have similar sequence and structure but fold independently, and by different pathways. The C-terminal domain alone is unstable under acidic conditions but exhibits a high degree of stability in acid environments when complexed with its binding partner the N-terminal domain [45]. This suggests that acid instability in one binding partner can be abrogated through interaction with an acid-stable partner and the instability of the CH1 domain may therefore be rescued by the high stability of the CL domain. Interestingly, results from the literature suggest that engineering the CH1 domain to contain more disulphide bonds can increase the thermal stability of the Fab [46]. The interactions between the CH1 and CL and the VH and VL appear to be important, and may rescue the less stable CH1 domain, but there appears to be little stabilisation between the CH-CL and VH-VL dimers [44], although the CH-CL dimer may play a role in desolvating hydrophobic moieties on the VH-VL dimer that are exposed when the VH and VL are expressed as a scFv [47].

**Fig. 3:**
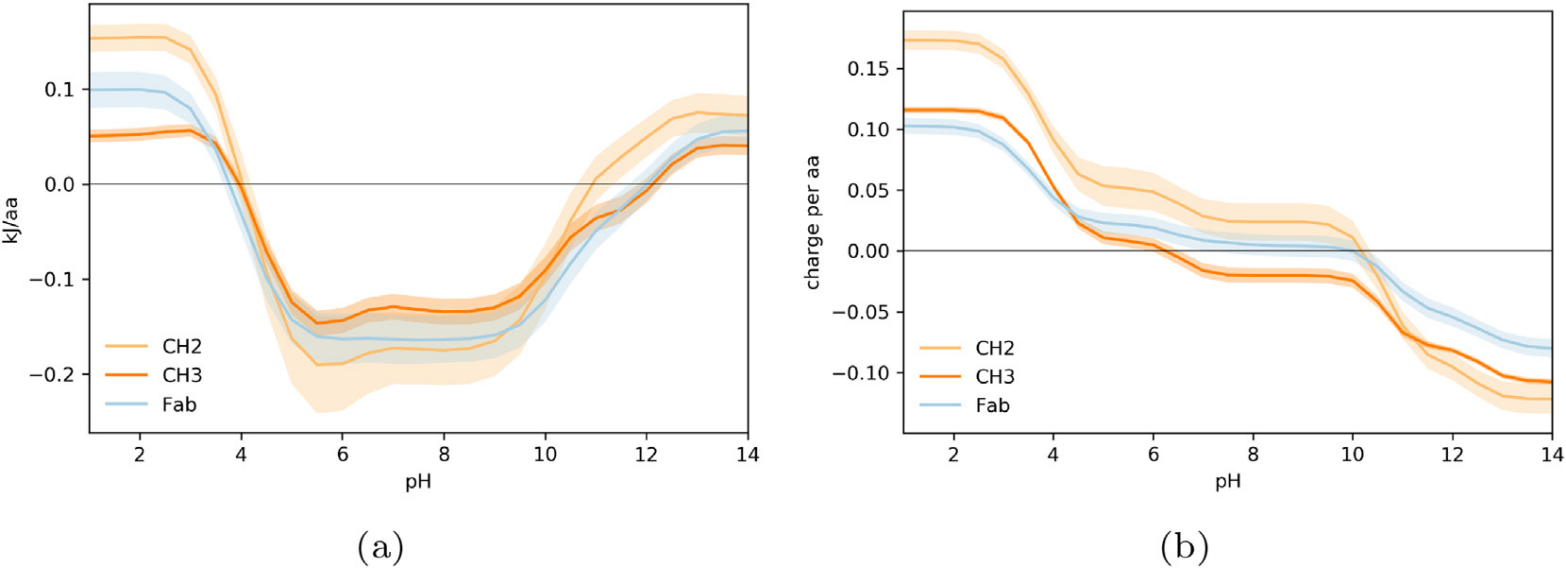
Calculated pH-dependence of folded state energy and charge for Fab and Fc domains. (a) The pH-dependent of folded state energy shown for Fab compared with Fc domains. (b) Predicted pH-titrated charge.

After investigating the Fab, we then moved on to compare the Fab as a whole to the CH2 and CH3 domains of the Fc, since there are pH-dependent experimental data with which to compare for these constituents (Fig. 3). For example, results in the literature suggest that the Fab has a lower melting temperature than the Fc [48], [49], [50], however the Fc is believed to unfold faster than the Fab in the presence of chemical denaturants [51], and the Fc is more sensitive than the Fab to aggregation at low pH [27], [49]. In terms of ionisable contributions to the folded state stability (Fig. 3a), the CH2 domain appears to be more stable than the CH3 domain at neutral pH, but less stable at low pH. The Fab substructure lies between the CH2 and CH3 domains in terms of stability at both neutral and low pH. Notably, out of all of the IgG domains, the CH2 domain appears to be the domain most stabilised by electrostatic contributions at physiological pH, but is amongst the most destabilised at low pH. This is to be expected as proteins with a greater degree of charge stabilisation at neutral pH will be disproportionately affected once these charge groups are neutralised, and therefore proteins that are highly stabilised at neutral pH are more likely to show instability through acid pH titration. From our calculations, the CH2 domain also exhibits the greatest magnitude of charge, as well as the most positive charge at low pH, and is amongst the most charged at physiological pH which may explain both the high level of charge-charge stability at neutral pH, but also the relative instability at low pH (Fig. 3b).

Our understanding is that the pH instability of the CH2 domain is in part related to its high stability at neutral pH, and the importance of the CH2 domain in acid aggregation has also been observed experimentally. The role of the CH2 domain for driving IgG aggregation in an acidic environment appears to be linked to the glycosylation state of the CH2 domain, with aglycosylated mAbs demonstrating a lower T_m_ [52], [27], [53] and fragmentation of the IgG at pH 4 also appears to occur in the CH2 domain [54]. Also upon exposure to low pH, the CH2 appears to undergo a structural transformation and once renatured by neutral pH titration, leads to an increased thermal stability of 12-15°C for the whole IgG [55], however this conformation change appears to have little effect on the structure of the IgG overall [56]. Subsequent work from the same group suggests that before acid treatment, the CH2 exhibits stronger interactions with the CH3 domain, but upon renaturation, the CH2 instead forms stronger interactions with the CH1 domain [57]. Lastly, experimental determination of the conformational stability of isolated IgG constant domains using circular dichroism suggests that CH2 and CH3 domains are less stable than the CH1-CL dimer at pH 2 [58]. Our work suggests that of the Fc domains, both structures exhibit a relative sensitivity to acid pH, but as CH2 exhibits a more destabilising energy in the acidic regime we suggest CH2 will be more acid sensitive.

Thus, to alleviate the relative instability of the CH2 domain in the low pH region, it is not as simple as mutating charge interactions, as stability would then be compromised across a range of pH conditions. Instead, the interplay between the contribution from ionisable group interaction needs to be considered against other stabilising contributions to the folded state. To better understand the importance of the electrostatic stability contribution, and the differences between the apparent relative acid stability of the CH1 and the instability of the CH2, we investigated the electrostatic contribution to stability at low pH for proteins that have evolved for stability at low pH.

### 3.2 Stomach resident proteases have reduced ionisable charge pair interactions

**Fig. 4:**
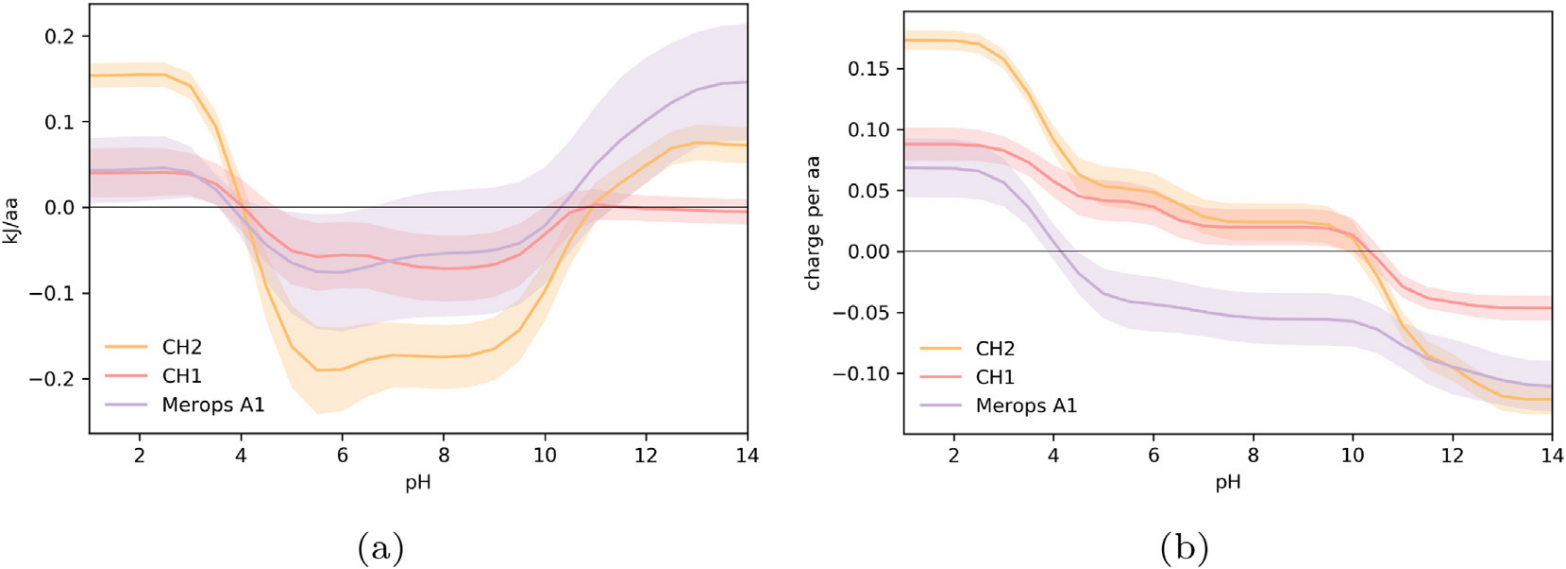
Comparison of calculated pH-dependence for Fc domains and acid-stable proteases. (a) pH-dependence of folded state energy. (b) pH-titrated charge.

In order to contextualise the apparent pH instability of the IgG CH2 domain, and relative stability of the CH1 domain, we compared both IgG domains to proteins that have evolved to low pH environments. In Fig. 4 we compare the CH1 and CH2 to the Merops A1 family of stomach resident aspartic proteases. The IDP-like CH1 and the acid stomach proteins appear to be less destabilised by the transition to the low pH regime, and are thus less likely to unfold. In comparison, the CH2 domain, which is believed to be acid sensitive but stable at physiologically pH, has a markedly different charge contribution profile to the acid-stable proteins. The CH1 domains and the stomach proteases have reduced ionisable group interactions compared to the CH2 domains, and therefore have less stabilisation to lose upon D/E neutralisation at the low pH found in the stomach (pH 1.5–3.5).

**Fig. 5:**
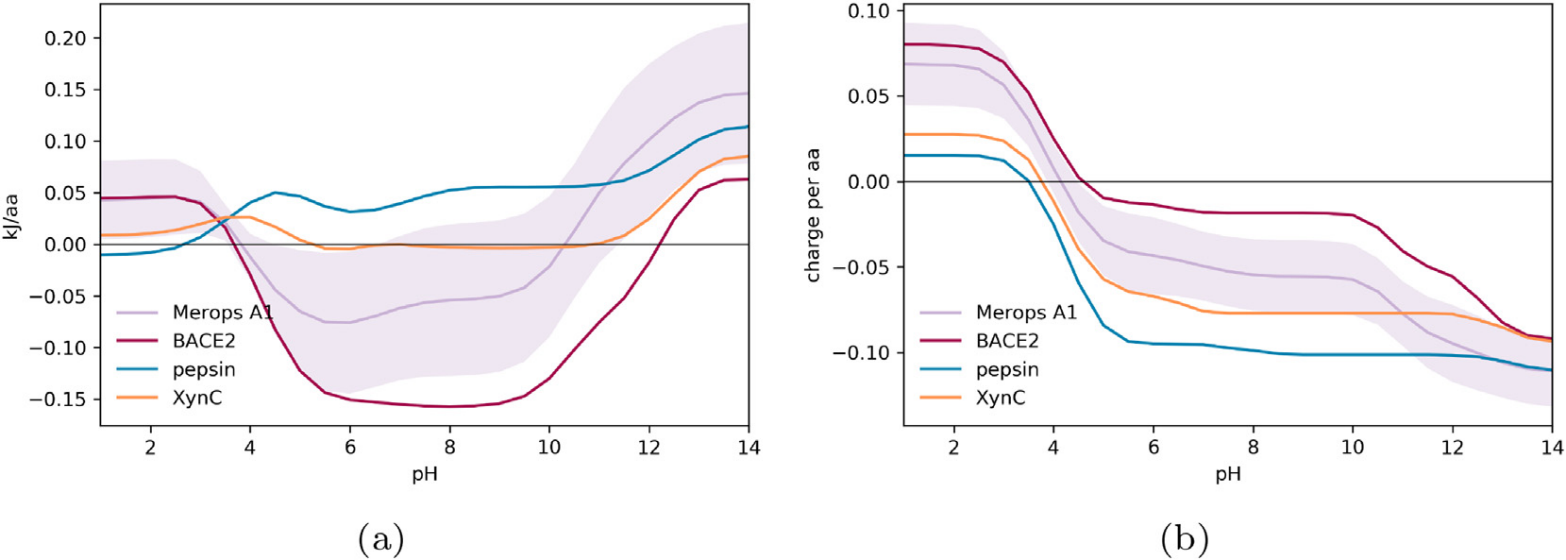
Comparison of calculated pH-dependence for proteins with differing acid-stability. (a) pH-dependence of folded state. (b) pH-titrated charge.

Next, we calculated the ionisable energy contribution to folded state stability for a pepsin, a specific acid-stable protease and BACE2, a (human) homologue of pepsin that is not stomach resident. For comparison we also calculated the energy and charge profiles for a industrially important xylanase C (XynC) enzyme from the acidophile *Aspergillus kawachii* (Fig. 5) which has an optimum pH of 2 for catalytic activity but is stable at pH 1, and in comparison to mesophillic xylanase C proteins, lacks the characteristic Ser/Thr surface in favour of expressing a negative surface with anisotropically distributed acidic residues [38].

BACE2 is predicted to have a larger contribution from ionisable group interactions, and thus stability, at neutral pH, which is lost at acidic pH (Fig. 5a). By contrast, the pH-dependence for pepsin and XynC are much flatter so that although the ionisable group interactions do not contribute greatly to fold stability at neutral pH, they do not lead to acid instability. In terms of charge (Fig. 5b), pepsin and XynC are clearly highly acidic proteins. Charged protein surfaces are maintained in acid-stable proteins, but they are not necessarily used extensively in the formation of salt-bridges. Thus, although pH-dependence is flattened, consistent with evolution for an acid environment, the question remains of how charged group interactions are arranged so as to maintain stability at neutral pH.

### 3.3 Pepsin comparison with BACE2 homologue reveals repurposing of acidic sidechains from salt bridges to hydrogen-bonding interactions

**Fig. 6:**
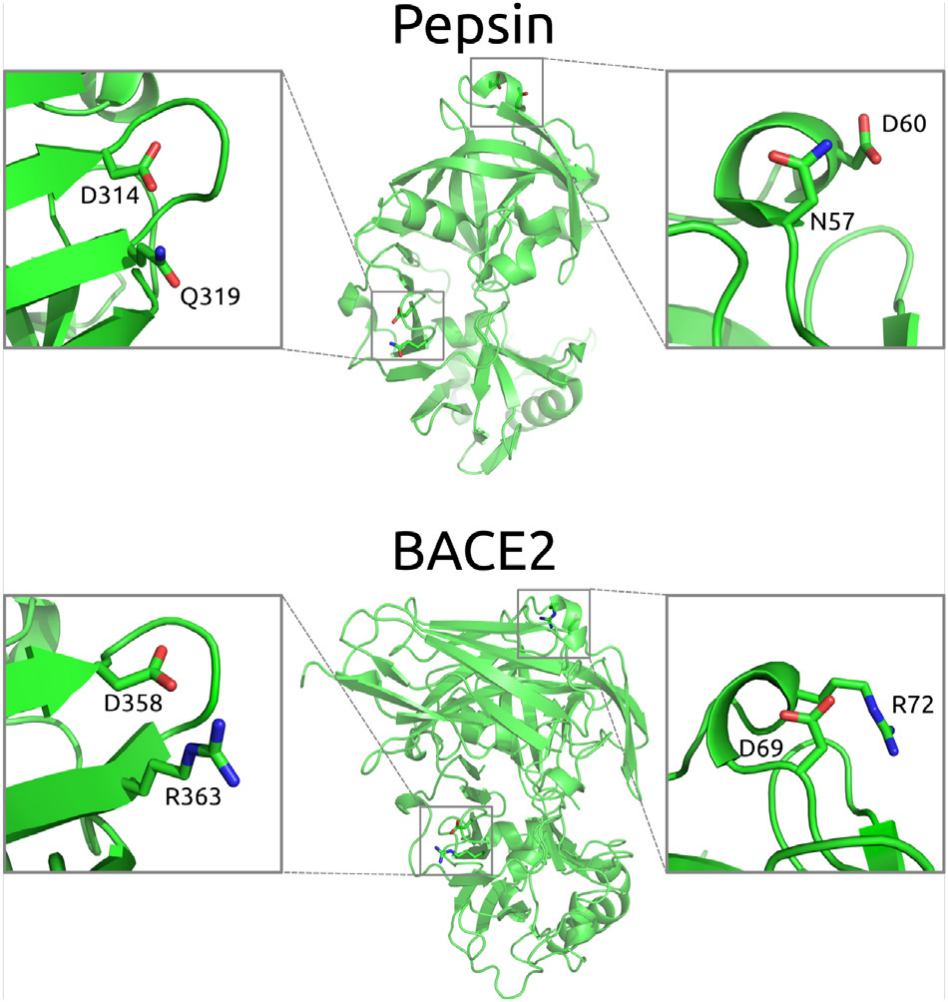
Comparison of acidic group interactions in equivalent regions of the homologues pepsin and BACE2.

In a closer look at how charge interactions are used for folded state stability at neutral and acidic pHs, specific examples are identified graphically for pepsin and its BACE2 homologue. Fig. 6 shows two examples where basic amino acids in BACE2 have been replaced by amino acids with polar sidechains in pepsin. In each case, interaction with an acidic sidechain is maintained, but rather than positive–negative charge pairing, this is now hydrogen-bonding. We infer that at the acidic pH of the stomach, where a positive charge would be left unpaired as a neighbouring acidic group protonates and neutralises, a polar but uncharged sidechain allows for formation of a hydrogen-bond network that can adapt and be maintained through protonation of the neighbouring acidic group.

In the case of the D358-R363 interaction in BACE2, a glutamine replaces the arginine in the D314-Q319 equivalent pairing for pepsin. In both BACE2 and pepsin, the aspartic acid sidechains also make hydrogen bonds to mainchain groups, presumably contributing to overall fold stability, at least locally in the loop region displayed. Also shown in Fig. 6, the D69-R72 charge pair in BACE2 is replaced by the D60-N57 hydrogen bond pair in pepsin. In this case, although structurally equivalent, the relative locations are inverted between the two enzymes.

In general, it appears that pepsin can maintain a stabilising network of negative charges through swapping in hydrogen-bond interactions for charge pair and salt-bridge interactions, giving a basis for redesign of surface charges in domains that are required to be stable at low pH, but have not evolved with that restraint.

**Fig. 7:**
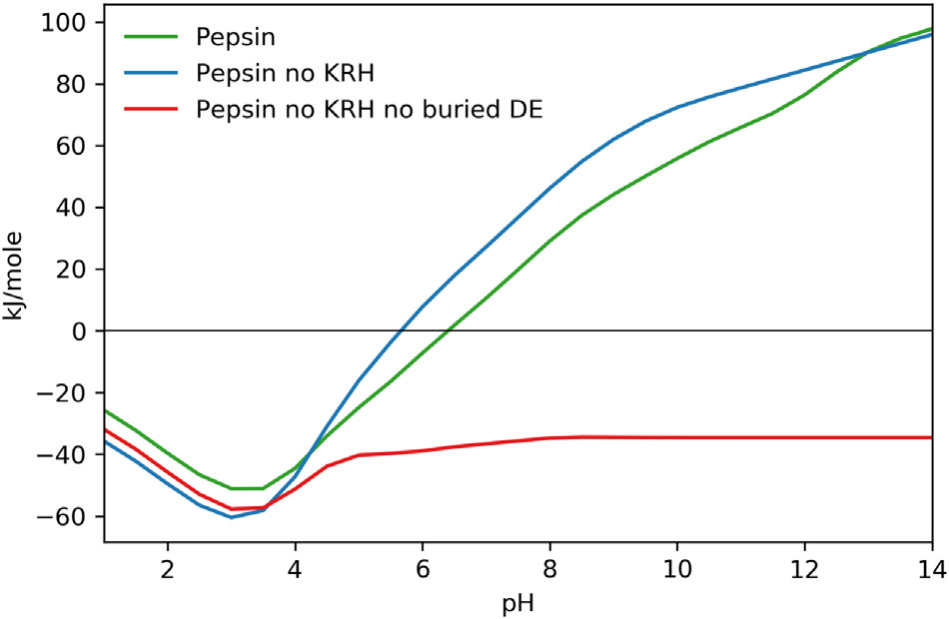
FDDH calculations of pH-dependence for human pepsin. Unaltered pepsin (green) is shown alongside pepsin with all basic amino acids mutated to non-polar amino acids blue), and with the further mutation of buried acidic amino acids to non-polar groups (red).

In order to show calculations of pH-dependent stability that are accessible with the heat map plots at www.protein-sol.manchester.ac.uk [42], the Debye-Hückel (DH) method was used. Previous work shows that this accounts reasonably well for interactions between ionisable groups that are on the protein surface [31], whereas buried and partially buried groups are better analysed with a combined Finite Difference Poisson–Boltzmann (FDPB) and DH methodology, termed FDDH [41]. The FDDH method includes better estimation of hydrogen-bonding energetics to ionisable groups in less solvent accessible regions, generally the type of interactions that we have identified in pepsin (Fig. 6). Since FDDH calculations are more computationally expensive than DH, they are not currently available on the protein-sol web server, but local computations for human pepsin and BACE2 pH-dependence have been made. For most proteins, DH and FDDH calculations give (qualitatively at least) a similar predicted pH-dependence of folded state stability, since buried ionisable group interactions do not play the major role in establishing this dependence. This similarity is also the case for BACE2 (Supp Fig. 1). However, the DH (Fig. 5) and FDDH curves (Fig. 7) human pepsin are quite different in two notable ways. First, there is a steep pH-dependence between pH 4 and alkaline pH for the FDDH calculations, that is absent for DH, and which is a sharp deviation from most proteins, giving a clear tendency towards maximal stability at acidic pH. The reducing stability as pH rises from acid pH is due to buried aspartic and glutamic acid sidechains that have elevated pKas, as noted previously for (non–human) pepsin [59]. We demonstrate (Fig. 7) that this variation is not removed by calculation with complete removal of basic sidechains (mutation of Lys and Arg to Met and His to Leu in 3utl), but is absent with the additional mutation (to Leu) of 5 buried acidic groups in 3utl (D96, E107, D149, D215, D303). Only one of these groups (D215) is part of the specific aspartic acid catalytic machinery, so most of the effect originates from outside of the active site. Second, and equally significant, is that even with the complete absence of basic sidechains, through modelled mutation, pepsin still exhibits a significant stabilisation of the folded form at pH 3 (Fig. 7). This must arise from hydrogen-bond interactions to acidic groups, consistent with the exemplar graphical observations shown in Fig. 6, and evidence for a systematic mechanism for using acidic groups to specifically stabilise protein folds in pH regions where stability is lost through neutralisation of salt-bridges.

### 3.4 Immunoglobulin superfamily domains also repurpose acidic sidechain interactions for acid tolerance

Having established hypotheses for how proteins might evolve to maintain folded state stability at low pH, we sought to further explore the link with immunoglobulin superfamily (IgSF) domains. Although the CH3 and CH2 domains of IgGs have different stabilities to low pH, neither has evolved to be stable in an acidic environment where aspartic and glutamic acid sidechains are neutralised. IgSF domains in *E. coli* have been reviewed [60], for which some are cell surface and therefore may be expected to experience the low pH of environments where the bacteria colonise. We looked closely at the IgSF domain of the FimA protein, for which a structure is available (PDB id2jty [61]). The ‘heat map’ tool of the protein-sol server [42] was used to compare CH2 and CH3 domains of an IgG (PDB id1hzh), and the immunoglobulin domain of FimA (SI Fig. 2, panels A, B, C). The reduced pH-dependent contribution to stability of CH3 compared with CH2 (Fig. 2) is replicated in these plots of predicted stability versus pH and ionic strength. Strikingly, FimA has yet lower pH-dependence of stability, reminiscent of pepsin (Fig. 5).

Observing that IgSF domains can be also pushed towards lower pH-dependence of stability, when subjected to evolutionary constraints, we looked further at whether the mechanisms were similar to those seen for pepsin (in comparison with BACE2). The percentages of charged residues (at neutral pH: D, E, K, R, H) are 28% (CH2), 21% (CH3) and just 13% (FimA). Further, the values for basic amino acids are 12% (CH2), 9% (CH3) and 4% (FimA). Thus basic residues in particular are selected against, consistent with avoiding the deleterious effect on stability as they lose acidic group partners upon neutralisation at low pH. The question remains of how acidic groups are organised so as to contribute to folded state stability at neutral and very acidic pH values. Other than one network between acidic residues and a histidine, FimA eschews salt-bridge type interactions. In their place, the carboxylate to polar (but uncharged) sidechain interactions highlighted in pepsin are evident, as well as hydrogen bonds to mainchain groups. It appears that similar mechanisms are used by nature to evolve a reduced pH-dependent stability of the folded state, and acid-tolerance, across the disparate families of aspartic acid proteases and proteins that contain immunoglobulin domains.

Although FimA contains the immunoglobulin domain, it is far removed from the Ig domains of antibodies. Structural alignment of FimA to CH2 or CH3 gives multiple solutions for different registers of β-strands. Nevertheless, it is useful to examine the location of interactions around carboxylate groups in FimA (Figure SI 2, panels D, E, F), which can be transferred to similar sites in the Ig domains of antibodies (such as CH2), for potential engineering towards acid-tolerance. According to our pKa calculations (underlying the predictions of pH-dependence), the 3 sites of largest interaction in FimA are around D24, D62, and D105. In one case hydrogen bond interactions are to polar sidechains from amino acids that are distant in sequence (D24, Figure SI 2 panel E), whilst in the other two aspartic acid interacts with both polar sidechain and mainchain hydrogen-bonding groups, and across loops connecting neighbouring β-strands (Figure SI 2, panel F/ D62, panel G/ D105).

Predictions of pH-dependence are made with ionisable group interactions in the unfolded state set to zero. This approximation is used due to a lack of detailed knowledge of structure in the unfolded state, and therefore inability to make accurate structure-based calculations of the contribution of the unfolded state to folding stability. It is clear though that pKas can perturbed from model compound values by interactions in the unfolded state [62]. Our own work in this area trialled a simple model of charge interactions in the unfolded state scaling according to sequence separation [31]. Even this simple estimate generally improved agreement with experiment, but to a variable extent and in all cases studied leaving the form of pH-dependence unchanged (relative to omission of the unfolded state model). The predictions made in this study point to a systematic swap of interaction environment for acidic sidechains between proteins evolved to function at neutral and at very low pH values. This is essentially a qualitative result, but one that can inform protein engineering for acid tolerance, as well as leading to more detailed experimental analysis of the balance of interactions in charged and hydrogen bonding networks, between folded and unfolded states.

Another approximation made in our work is the neglect of glycan effects, due to the lack of consistent structural information over the datasets used in our calculations. Predictions of protein pKas and pH-dependence generally exclude glycosylation. Reports suggest that in addition to the potential for glycosylation to increase folded state stability at neutral pH, the effect can be pH and domain dependent [53], [52], [54], [63]. For example, it has been shown that glycosylation of CH2 increased the apparent Tm of an IgG1 Fc by about 5°C at pH 3.5, but only half of this at pH 7 [53]. The form of the pH-dependence remains the same between glycosylated and un-glycosylated CH2 variants. Our analysis is qualitative but leads to firm predictions for engineering acid tolerant proteins. It is clear though that detailed molecular understanding will need to incorporate other factors, including the role of glycosylation. One possible connection between our work and glycosylation effects is where acid protein sidechains interact with sugar hydroxyls, they could effectively be forming the type of network seen in pepsin and FimA.

## 4 Conclusions

The potential of therapeutic antibodies and next generation protein pharmaceuticals is well known, however issues remain with optimisation of production. In particular, the low pH hold required for viral inactivation and protein A chromatography leads to aggregation which can limit the development of therapeutic antibodies. We present a computational study of pH-dependence for IgG domains, the qualitative trends of which are in agreement with experimental data. The CH1 domain of the Fab domain exhibits similar charge-charge contributions to folded state stability at low pH as proteins that have evolved to reside in low pH environments. However, the similarity is limited, CH1 domains are not stable folded proteins in isolation, and thus mechanisms for designing in acid-stability are suggested that focus on proteins that have evolved to function at low pH. For the CH2 domain, the same groups that provide extensive stabilisation at neutral pH lead to destabilisation at acidic pH. This observation is consistent with experimental data showing that CH2 folded state stability is more acid sensitive than that of the CH3 domain, giving us confidence that the modelling can be used to consider engineering for acid stability. Comparison of proteins within the same family, but evolved to function at widely differing pH values leads to the suggestion that, in the low pH environment, basic sidechains (that are paired with acidic sidechains) can be swapped out for polar hydrogen-binding sidechains. Although ionisable group interactions are not the only stabilising force available to the protein, differences in stability at neutral pH and acidic pH are likely to be related to changes in the ionisation state of acidic and basic residues. A better understanding of these forces may provide insights for engineering acid-stable therapeutics in the future.

## Additional information

**Competing interests:** The authors declare that they have no competing interests.

## Funding

UK EPSRC grant EP/N024796/1.

## CRediT authorship contribution statement

**Max Hebditch:** Methodology, Software, Validation, Formal analysis, Investigation, Data curation, Writing - original draft, Writing - review & editing, Visualization. **Ryan Kean:** Formal analysis, Investigation, Data curation. **Jim Warwicker:** Conceptualization, Software, Validation, Formal analysis, Investigation, Data curation, Writing - original draft, Writing - review & editing, Visualization, Supervision, Project administration, Funding acquisition.

## References

[1] Birch J.R., Racher A.J. (2006). Antibody production. Adv Drug Deliv Rev.

[2] Duhamel R.C., Schur P.H., Brendel K., Meezan E. (1979). pH gradient elution of human IgG1, IgG2 and IgG4 from protein A-sepharose. J Immunol Methods.

[3] Gronemeyer P., Ditz R., Strube J. (2014). Trends in upstream and downstream process development for antibody manufacturing. Bioengineering (Basel, Switzerland).

[4] Ulmer N., Vogg S., Müller-Späth T., Morbidelli M. (2019). Purification of human monoclonal antibodies and their fragments. Methods Mol Biol.

[5] Shukla Abhinav A., Thömmes Jörg (2010). Recent advances in large-scale production of monoclonal antibodies and related proteins. Trends Biotechnol.

[6] Brorson K., Brown J., Hamilton E., Stein K.E. (2003). Identification of protein a media performance attributes that can be monitored as surrogates for retrovirus clearance during extended re-use. J Chromatogr A.

[7] Shukla A.A., Hubbard B., Tressel T., Guhan S., Low D. (2007). Downstream processing of monoclonal antibodies – application of platform approaches. J Chromatogr B.

[8] Liu H.F., Ma J., Winter C., Bayer R. (2010). Recovery and purification process development for monoclonal antibody production. mAbs.

[9] Mazzer A.R., Perraud X., Halley J., O’Hara J., Bracewell D.G. (2015). Protein A chromatography increases monoclonal antibody aggregation rate during subsequent low pH virus inactivation hold. J Chromatogr A.

[10] McMahon M.J., O’Kennedy R. (2000). Polyreactivity as an acquired artefact, rather than a physiologic property, of antibodies: evidence that monoreactive antibodies may gain the ability to bind to multiple antigens after exposure to low pH. J Immunol Methods.

[11] Dimitrov J.D., Planchais C., Kang J., Pashov A., Vassilev T.L., Kaveri S.V., Lacroix-Desmazes S. (2010). Heterogeneous antigen recognition behavior of induced polyspecific antibodies. Biochem Biophys Res Commun.

[12] Ghose S., Allen M., Hubbard B., Brooks C., Cramer S.M. (2005). Antibody variable region interactions with Protein A: implications for the development of generic purification processes. Biotechnol Bioeng.

[13] Gülich Susanne, Uhlén Mathias, Hober Sophia (2000). Protein engineering of an IgG-binding domain allows milder elution conditions during affinity chromatography. J Biotechnol.

[14] Hober S., Nord K., Linhult M. (2007). Protein A chromatography for antibody purification. J Chromatogr B.

[15] Liu Z., Mostafa S.S., Shukla A.A. (2015). A comparison of protein a chromatographic stationary phases: performance characteristics for monoclonal antibody purification. Biotechnol Appl Biochem.

[16] Shukla A.A., Hinckley P. (2008). Host cell protein clearance during protein a chromatography: development of an improved column wash step. Biotechnol Progr.

[17] Hanke A.T., Ottens M. (2014). Purifying biopharmaceuticals: knowledge-based chromatographic process development. Trends Biotechnol.

[18] Chen C., Wakabayashi T., Muraoka M., Shu F., Wei Shan C., Chor Kun C., Tim Jang C., Soehano I., Shimizu Y., Igawa T. (2019). Controlled conductivity at low pH in Protein L chromatography enables separation of bispecific and other antibody formats by their binding valency. mAbs.

[19] Kanje S., Venskutonytė R., Scheffel J., Nilvebrant J., Lindkvist-Petersson K., Hober S. (2018). Protein engineering allows for mild affinity-based elution of therapeutic antibodies. J Mol Biol.

[20] Burnouf T., Radosevich M. (2003). Nanofiltration of plasma-derived biopharmaceutical products. Haemophilia.

[21] Klutz S., Lobedann M., Bramsiepe C., Schembecker G. (2016). Continuous viral inactivation at low pH value in antibody manufacturing. Chem Eng Process.

[22] Yamasaki H., Tsujimoto K., Koyama H., Ejima D., Arakawa T. (2008). Arginine facilitates inactivation of enveloped viruses. J Pharmaceutical Sci.

[23] Vázquez-Rey M., Lang D.A. (2011). Aggregates in monoclonal antibody manufacturing processes. Biotechnol Bioeng.

[24] Brummitt R.K., Nesta D.P., Chang L., Chase S.F., Laue T.M., Roberts C.J. (2011). Nonnative aggregation of an IgG1 antibody in acidic conditions: part 1. Unfolding, colloidal interactions, and formation of high-molecular-weight aggregates. J Pharmaceutical Sci.

[25] Brummitt R.K., Nesta D.P., Chang L., Kroetsch A.M., Roberts C.J. (2011). Nonnative aggregation of an IgG1 antibody in acidic conditions, part 2: nucleation and growth kinetics with competing growth mechanisms. J Pharmaceutical Sci.

[26] Arosio P., Rima S., Morbidelli M. (2013). Aggregation mechanism of an IgG2 and two IgG1 monoclonal antibodies at low pH: from oligomers to larger aggregates. Pharmaceutical Res.

[27] Liu B., Guo H., Xu J., Qin T., Xu L., Zhang J., Guo Q., Zhang D., Qian W., Li B. (2016).

[28] Imamura H., Honda S. (2016). Kinetics of antibody aggregation at neutral pH and ambient temperatures triggered by temporal exposure to acid. J Phys Chem B.

[29] Skamris T., Tian X., Thorolfsson M., Karkov H.S., Rasmussen H.B., Langkilde A.E., Vestergaard B. (2016). Monoclonal antibodies follow distinct aggregation pathways during production-relevant acidic incubation and neutralization. Pharmaceutical Res.

[30] Imamura H., Sasaki A., Honda S. (2017). Fate of a stressed therapeutic antibody tracked by fluorescence correlation spectroscopy: folded monomers survive aggregation. J Phys Chem B.

[31] Warwicker J. (1999). Simplified methods for pKa and acid pH-dependent stability estimation in proteins: removing dielectric and counterion boundaries. Protein Sci.

[32] Hebditch M., Curtis R., Warwicker J. (2017). Sequence composition predicts immunoglobulin superfamily members that could share the intrinsically disordered properties of antibody ch1 domains. Sci Rep.

[33] Saphire E.O., Parren P.W.H.I., Pantophlet R., Zwick R.A., Morris G.M., Rudd P.M., Dwek R.A., Stanfield R.L., Wilson I.A. (2001). Crystal structure of a neutralizing human IgG against HIV-1: a template for vaccine design. Science.

[34] Berman H., Henrick K., Nakamura H., Markley J.L. (2007). The worldwide Protein Data Bank (wwPDB): ensuring a single, uniform archive of PDB data. Nucl Acids Res.

[35] Sievers F., Wilm A., Dineen D., Gibson T.J., Karplus K., Li W., Lopez R., McWilliam H., Remmert M., Söding J. (2011). Fast scalable generation of high-quality protein multiple sequence alignments using clustal omega. Mol Syst Biol.

[36] Rawlings N.D., Barrett A.J., Bateman A. (2011). Merops: the database of proteolytic enzymes, their substrates and inhibitors. Nucl Acids Res.

[37] Bailey D., Carpenter E., Coker A., Coker S., Read J., Jones A.T., Erskine P., Aguilar C., Badasso M., Toldo L. (2012). An analysis of subdomain orientation, conformational change and disorder in relation to crystal packing of aspartic proteinases. Acta Crystallogr, Sect D: Biol Crystallogr.

[38] Fushinobu S., Ito K., Konno M., Wakagi T., Matsuzawa H. (1998). Crystallographic and mutational analyses of an extremely acidophilic and acid-stable xylanase: biased distribution of acidic residues and importance of Asp37 for catalysis at low pH. Protein Eng.

[39] Banner D.W., Gsell B., Benz J., Bertschinger J., Burger D., Brack S., Cuppuleri S., Debulpaep M., Gast A., Grabulovski D. (2013). Mapping the conformational space accessible to BACE2 using surface mutants and cocrystals with Fab fragments, Fynomers and Xaperones. Acta Crystallogr Section D: Biol Crystallogr.

[40] Moutevelis E., Warwicker J. (2004). Prediction of pKa and redox properties in the thioredoxin superfamily. Protein Sci.

[41] Warwicker J. (2004). Improved pKa calculations through flexibility based sampling of a water-dominated interaction scheme. Protein Sci.

[42] Hebditch M., Warwicker J. (2019). Web-based display of protein surface and pH-dependent properties for assessing the developability of biotherapeutics. Sci Rep.

[43] Vanhove M., Usherwood Y.K., Hendershot L.M. (2001). Unassembled Ig heavy chains do not cycle from BiP in vivo but require light chains to trigger their release. Immunity.

[44] Röthlisberger D., Honegger A., Plückthun A. (2005). Domain interactions in the Fab fragment: a comparative evaluation of the single-chain Fv and Fab format engineered with variable domains of different stability. J Mol Biol.

[45] Mayr E.-M., Jaenicke R., Glockshuber R. (1997). The domains in *γ*b-crystallin: identical fold-different stabilities. J Mol Biol.

[46] Peters S.J., Smales C.M., Henry A.J., Stephens P.E., West S., Humphreys D.P. (2012). Engineering an improved IgG4 molecule with reduced disulfide bond heterogeneity and increased fab domain thermal stability. J Biol Chem.

[47] Nieba L., Honegger A., Krebber C., Plückthun A. (1997). Disrupting the hydrophobic patches at the antibody variable/constant domain interface: improved in vivo folding and physical characterization of an engineered scFv fragment. Protein Eng.

[48] Welfle K., Misselwitz R., Hausdorf G., Höhne W., Welfle H. (1999). Conformation pH-induced conformational changes, and thermal unfolding of anti-p24 (HIV-1) monoclonal antibody CB4-1 and its Fab and Fc fragments, Biochimica et Biophysica Acta (BBA)-Protein Structure and Molecular. Enzymology.

[49] Vermeer A.W., Norde W. (2000). The thermal stability of immunoglobulin: unfolding and aggregation of a multi-domain protein. Biophys J.

[50] Vermeer A.W., Norde W., van Amerongen A. (2000). The unfolding/denaturation of immunogammaglobulin of isotype 2b and its Fab and Fc fragments. Biophys J.

[51] Rowe E.S. (1976). Dissociation and denaturation equilibria and kinetics of a homogeneous human immunoglobulin Fab fragment. Biochemistry.

[52] Hari S.B., Lau H., Razinkov V.I., Chen S., Latypov R.F. (2010). Acid-induced aggregation of human monoclonal IgG1 and IgG2: molecular mechanism and the effect of solution composition. Biochemistry.

[53] Latypov R.F., Hogan S., Lau H., Gadgil H., Liu D. (2012). Elucidation of acid-induced unfolding and aggregation of human immunoglobulin IgG1 and IgG2 Fc. J Biol Chem.

[54] Gaza-Bulseco G., Liu H. (2008). Fragmentation of a recombinant monoclonal antibody at various pH. Pharmaceutical Res.

[55] Martsev S.P., Kravchuk Z.I., Vlasov A.P. (1994). Large increase in thermal stability of the CH2 domain of rabbit IgG after acid treatment as evidenced by differential scanning calorimetry. Immunol Lett.

[56] Ejima D., Tsumoto K., Fukada H., Yumioka R., Nagase K., Arakawa T., Philo J.S. (2007). Effects of acid exposure on the conformation, stability, and aggregation of monoclonal antibodies. Proteins: Struct, Funct, Bioinf.

[57] Martsev S.P., Kravchuk Z.I., Vlasov A.P., Lyakhnovich G.V. (1995). Thermodynamic and functional characterization of a stable IgG conformer obtained by renaturation from a partially structured low pH-induced state. Febs Lett.

[58] Yageta S., Lauer T.M., Trout B.L., Honda S. (2015). Conformational and colloidal stabilities of isolated constant domains of human immunoglobulin G and their impact on antibody aggregation under acidic conditions. Mol Pharm.

[59] Lin X., Loy J.A., Sussman F., Tang J. (1993). Conformational instability of the n-and c-terminal lobes of porcine pepsin in neutral and alkaline solutions, protein. Science.

[60] Bodelόn Gustavo, Palomino Carmen, Fern ández Luis Ángel (2013).

[61] Puorger Chasper, Vetsch Michael, Wider Gerhard, Glockshuber Rudi (2011). Structure, folding and stability of FimA, the main structural subunit of type 1 pili from uropathogenic Escherichia coli strains. Journal of molecular biology. Elsevier.

[62] Tan Yee-Joo, Oliveberg Mikael, Davis Ben, Fersht Alan R. (1995). Perturbed pKA-values in the Denatured States of Proteins. Journal of Molecular Biology. Elsevier.

[63] Zheng Kai, Yarmarkovich Mark, Bantog Christopher, Bayer Robert, Patapoff Thomas W. (2014). Influence of glycosylation pattern on the molecular properties of monoclonal antibodies. MAbs.

