# Supplementary material for: Modelling of pH-dependence to develop a strategy for stabilising mAbs at acidic steps in production
Source: Comput Struct Biotechnol J. 2020 Mar 10;18:897–905. doi: 10.1016/j.csbj.2020.03.002 (PMC7171260; doi:10.1016/j.csbj.2020.03.002)
Supplement: Supplementary file 1 [file mmc1.zip › Hebditch-Kean-Warwicker2019-SI.pdf]

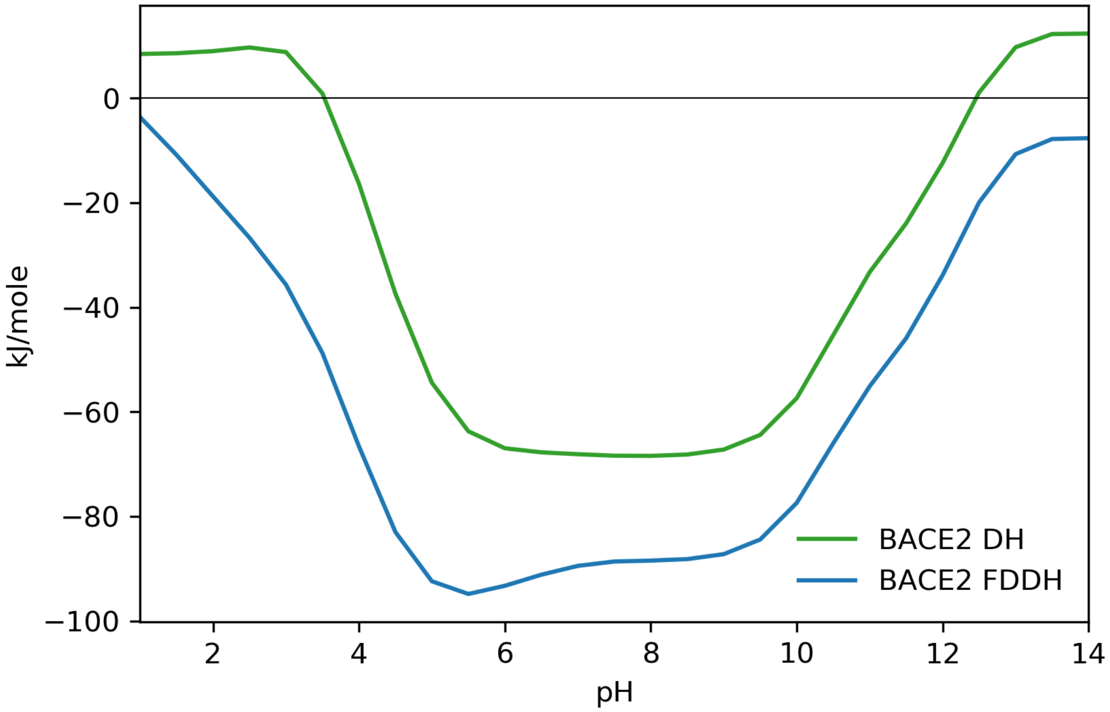

Figure S1: Comparison of acidic group interactions in equivalent regions of the homologues pepsin and BACE2.

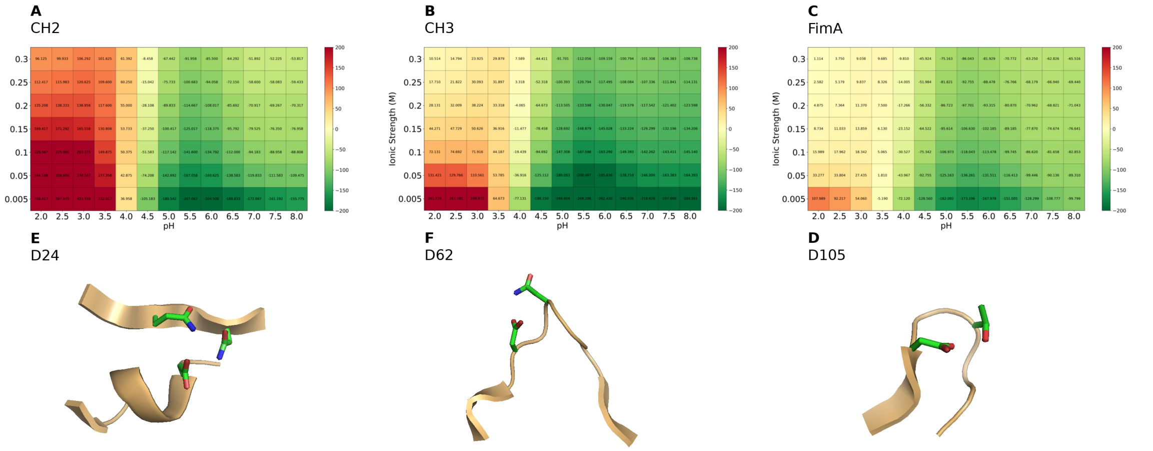

Figure S2: pH-dependence in immunoglobulin superfamily domains. Predictions of pH and ionic strength dependence of folded state stability are made with the ‘heat map’ option at www.protein-sol.manchester.ac.uk/heatmap, for the CH2 (panel A) and CH3 (panel B) domains of 1hzh, and FimA (2jty, panel C). Lower gradation in colour across the pH-range indicates lower stability change upon pH variation. Units for the energy scale bar are J/mole per amino acid, so that protein size is factored out. Three locations of greatest predicted pKa change are shown for ionisable groups in FimA. All are hydrogen bonding networks, rather than salt-bridge interactions. D24 hydrogen bonds with the sidechains of Q57 and N59 (panel D); D62 hydrogen bonds to the sidechain of N64 and into the two mainchain peptide groups surrounding N64 (panel E); D105 interacts with the sidechain of T107 and the mainchain flanking this sidechain.
